# Insights into the Complex Biological Network Underlying Myalgic Encephalomyelitis/Chronic Fatigue Syndrome

**DOI:** 10.3390/ijms27010268

**Published:** 2025-12-26

**Authors:** Dobrina Dudova, Martina Bozhkova, Steliyan Petrov, Ralitsa Nikolova, Teodora Kalfova, Mariya Ivanovska, Katya Vaseva, Maria Nikolova, Ivan N. Ivanov

**Affiliations:** 1 Department of Medical Microbiology and Immunology, Medical University of Plovdiv, 4002 Plovdiv, Bulgaria; dobrina.dudova@mu-plovdiv.bg (D.D.); steliyan.petrov@mu-plovdiv.bg (S.P.); ralitsa.nikolova@mu-plovdiv.bg (R.N.); teodora.kalfova@mu-plovdiv.bg (T.K.); mariya.ivanovska@mu-plovdiv.bg (M.I.); katya.vaseva@mu-plovdiv.bg (K.V.); 2 Research Institute, Medical University of Plovdiv, 4002 Plovdiv, Bulgaria; 3 National Reference Laboratory of Immunology, National Centre of Infectious and Parasitic Diseases, 1504 Sofia, Bulgaria; mr_nklv@yahoo.com; 4 Department of Microbiology, National Centre of Infectious and Parasitic Diseases, 1504 Sofia, Bulgaria; ivanoov@gmail.com

**Keywords:** myalgic encephalomyelitis, chronic fatigue syndrome, immune dysregulation, gastrointestinal tract dysbiosis, neuroendocrine alterations, mitochondrial dysfunction, endothelial dysfunction

## Abstract

Myalgic encephalomyelitis/chronic fatigue syndrome (ME/CFS) is a debilitating multisystem disorder characterized by immune dysregulation, metabolic impairments, neuroendocrine disturbances, endothelial dysfunction, and gastrointestinal abnormalities. Immune alterations include reduced natural killer cell cytotoxicity, T-cell exhaustion, abnormal B-cell subsets, and the presence of diverse autoantibodies, suggesting an autoimmune component. Gut dysbiosis and increased intestinal permeability may promote systemic inflammation and contribute to neurocognitive symptoms via the gut–brain axis. Neuroendocrine findings such as hypothalamic–pituitary–adrenal (HPA) axis hypofunction and altered thyroid hormone metabolism further compound metabolic and immune abnormalities. Metabolomic and mitochondrial studies identify impaired ATP generation, redox imbalance, and compensatory shifts toward alternative energy pathways underlying hallmark symptoms like post-exertional malaise. Endothelial dysfunction driven by oxidative and nitrosative stress, along with autoantibody-mediated receptor interference, may explain orthostatic intolerance and impaired perfusion. Collectively, ME/CFS appears to arise from a self-sustaining cycle of chronic inflammation, metabolic insufficiency, and neuroimmune imbalance.

## 1. Introduction

Myalgic encephalomyelitis/chronic fatigue syndrome (ME/CFS) is a complex, debilitating, and heterogeneous disorder. Despite decades of investigation, the etiology and pathogenesis of ME/CFS are not completely understood [1]. Proposed triggers include acute or chronic viral infections, reactivation of latent pathogens, significant physiological or psychological stressors, and genetic susceptibility. The current findings position ME/CFS as a multisystem disorder arising from the convergence of immune dysregulation, gastrointestinal dysfunction, neuroendocrine disturbances, metabolic impairments, and vascular pathology [2]. This review synthesizes current evidence across these interconnected biological domains and highlights key gaps that must be addressed to improve diagnostic and prognostic accuracy.

## 2. Definition

**Figure 1:**
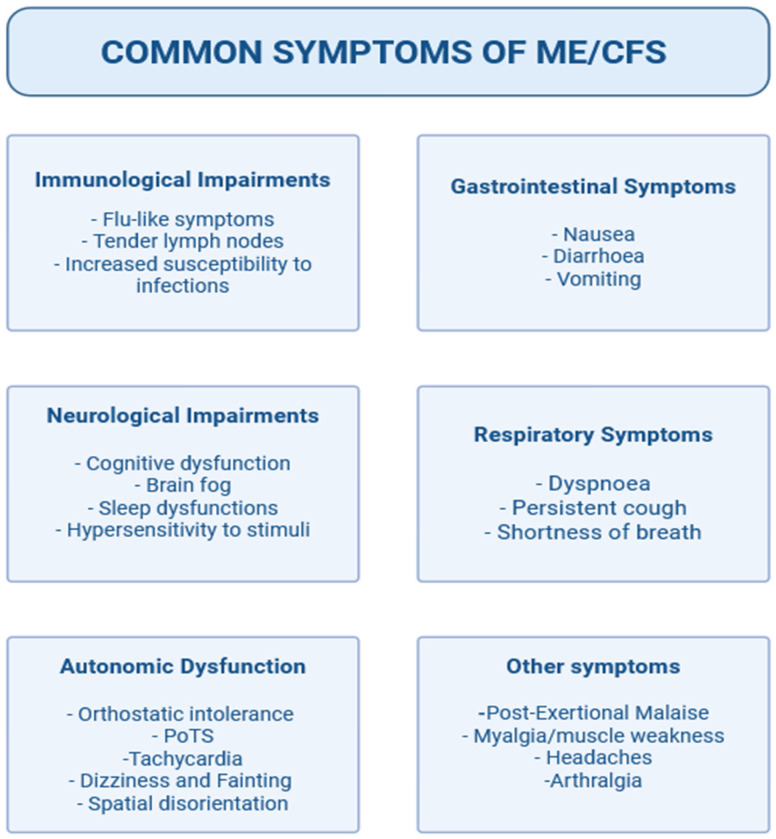
Summary of the most common symptoms of ME/CFS patients.

ME/CFS is medical condition characterized by persistent or recurring symptoms for more than 6 months, including fatigue, headaches, myalgia, arthralgia, post-exertional malaise, and sleep and cognitive dysfunction (Figure 1) [1].

The definition of ME/CFS has continuously evolved throughout the decades. Various disorders with similar symptoms were first mentioned at least 200 years ago, including neurasthenia, epidemic encephalomyelitis, atypical poliomyelitis, benign myalgic encephalomyelitis, and post-viral fatigue [3]. The first diagnostic criteria for this condition were proposed by the CDC in 1988, and since then multiple different diagnostic criteria exist (Table 1).

The one proposed by the Institute of Medicine (IOM) in 2015 is based on the following three symptoms: 1. significant reduction or limitation in the ability to perform pre-illness levels of daily activities that persists for more than 6 months, accompanied by fatigue that is often profound, of recent or definite (not lifelong) onset, and is not substantially relieved by rest; 2. post-exertional malaise; 3. unrefreshing sleep. At least one of the following is also required: cognitive impairment or orthostatic intolerance [10]. The Canadian Consensus Criteria for ME/CFS published in 2003 includes five additional required symptoms: pain; neurological/cognitive, autonomic, neuroendocrine, and immune manifestations [8].

The different diagnostic criteria lead to inconsistencies in the number of reported cases worldwide. The estimated prevalence of the condition varies widely between the existing sources. However, it has been predicted that the number of ME/CFS cases is around 17–24 million. The affected demographic includes people of all races and ages [1]. Moreover, the tendency is that females are more affected than males (approx. 2:1), and it is more frequently observed in individuals aged between 30 and 50 years old [1].

## 3. Etiology

ME/CFS is multifaceted condition associated with abnormalities in the immune, endocrine, and neuroendocrine systems, as well as dysfunctions of the gastrointestinal tract, metabolism, and endothelium [2]. However, the development of ME/CFS is still an enigma. Various hypotheses have been proposed, but there is still no united vision on its pathogenesis.

One of the hypotheses regarding its etiology is the presence of chronic infection or the reactivation of a latent one. The viral agents associated with the disorder are the following: Epstein–Barr virus (EBV); human herpes virus (HHV) 6A, 6B, 7, and 8; human parvovirus B19; cytomegalovirus (CMV); Ross River virus; enteroviruses; retroviruses; coronaviruses (incl. SARS-CoV-2) (Table 2) [12,13].

Host infection by different pathogens can trigger autoimmunity through various mechanisms [14]. Some infectious antigens share enough similarities (amino acid sequences, highly conserved structures, etc.) with self-molecules, and in accordance with the antigen mimicry theory this can result in the development of autoreactivity [14,15]. Another mechanism that could lead to autoimmunity is the damaging of self-tissue caused either by the pathogen itself or the excessive immune response to it. The damaged cells then release self-antigens that are taken by the antigen-presenting cells (APCs) and presented to the adaptive arm of the immune system, resulting in a self-specific response [14].

The second theory links ME/CFS to a gut microbiome imbalance. The gut microbiome plays a key role in regulating the immune system and metabolism. Research shows that people with ME/CFS often have an imbalance of gut bacteria, which can lead to increased gut permeability and systemic inflammation [16]. Dysbiosis can affect the production of important metabolites and neurotransmitters that affect brain function and energy metabolism [17,18,19,20]. This may explain many of the symptoms associated with ME/CFS, including cognitive problems and chronic fatigue. 

The third hypothesis views metabolic disorders as a leading cause. Disturbances in cellular energy metabolism may underline the persistent fatigue experienced by patients with ME/CFS. There is evidence that these patients have abnormalities in the production of energy and its usage, such as mitochondrial dysfunction. This can lead to a build-up of metabolic waste and insufficient ATP production, which in turn leads to symptoms such as fatigue, muscle weakness, and cognitive impairment [21,22].

## 4. Immunopathology

ME/CFS is a highly heterogenous condition, and contradictory data regarding the involvement of the immune system have been published. This could be explained by the lack of uniform diagnostic criteria, small cohorts, and improper patient selection. Furthermore, the testing methods largely differ, and results vary depending on the severity of the condition and how recent the onset of symptoms is. However, increasing evidence shows that immune dysfunction is present in a large portion of ME/CFS patients (Table 3).

One of the most consistent immunological changes that is observed in a large number of studies is decreased NK cell activity, as well as variations in their phenotype [23]. Abnormalities in cytolytic proteins (perforin, granzyme A and B), degranulation marker CD107a, as well as impaired mitogen-activated protein kinase (MAPK) phosphorylation, and calcium (Ca^2+^) mobilization in patients, when compared with healthy controls (HCs), have been reported [23,24,25]. The downstream polarization of MAPK in addition to the increase in intracellular Ca^2+^ concentrations are essential for NK cells’ cytotoxicity, including cytolytic protein exocytosis [23]. Furthermore, the TRPM3 ion channel was associated with Ca^2+^ influx and NK cell dysfunctions [52]. Changes in the above-mentioned could greatly affect the ability of the NK cells to properly exert their function, leading to higher chances of persistent infection and chronic inflammation, which could be observed in ME/CFS patients (Table 3). However, there are papers reporting no significant changes in the phenotype and cytotoxicity of NK cells in ME/CFS patients compared to HCs [29,30,53].

A growing body of evidence suggests that cytotoxic CD8+ T cells play a key role in the immunological mechanisms underlying this condition. While studies in the 1990s reported increased numbers of CD8+ T cells [54,55], more recent ones describe no significant difference in the absolute numbers of cytotoxic T cells [29,53,56]. However, there are reports of dysfunctional CD8+ T cells and more precisely clonally exhausted ones, characterized by progressive loss of effector functions (IL-2, IFNγ, TNFα), sustained high levels of inhibitory receptor expression (PD-1, CTLA-4, Lag-3, TIGIT, etc.), metabolic abnormalities, decreased proliferation, etc. [26,27]. This type of T-cell dysfunction is often seen in chronic viral infections and is caused by persistent antigen stimulation. Increased plasma levels of IL-10, which are found in patients with ME/CFS, play a key role in the development and the maintenance of clonally exhausted CD8+ T cells [27,28]. In addition, the metabolic abnormalities associated with this type of dysfunction could be found in ME/CFS. Normally, naïve T cells primarily rely on fatty acid oxidation (FAO) for catabolic metabolism; however, after activation of the cell, the signaling pathways increase glycolysis and fatty acid synthesis, while also lowering FAO. In ME/CFS, T cells exhibit inhibited glycolysis and promotion of FAO compared to HCs [27]. Moreover, there is a loss of mitochondrial membrane potential of CD8+ T cells in patients, which is a common sign of exhausted cells. This initiates the build-up of reactive oxygen species (ROS) and toxic metabolites, further worsening the T-cell exhaustion [27]. Likewise, CD4+ T cells demonstrate a significant reduction in glycolysis (Table 3) [28]. These findings are consistent with the hypothesis that chronic viral infection could trigger the onset of ME/CFS.

Reports about regulatory T cells (Tregs) are also contradictory. With the exception of one study, most reports indicate a higher absolute count of Tregs in patients compared with healthy controls [29,30,31,32]. Tregs are a specialized subset of T lymphocytes that modulate immune responses. Sustained Treg deficiency and reduced activity in the context of chronic inflammation may exacerbate tissue damage, and it can also reflect or contribute to immune exhaustion [30]. On the other hand, increased Treg numbers or activity could lead to the suppression of effector T cells, NK cells, and other antiviral responses, resulting in failure of the immune system to clear latent viral infections, sustaining chronic immune dysregulation [57].

Similarly, the evidence about alterations in cytokine profiles is not consistent. A significant increase in Th2-like cytokines accompanied by suppression of Th1 and Th17 cytokines has been reported [37]. However, a mixed response with the expression of both Th1- and Th2-associated cytokines has also been shown (Table 3) [38]. Moreover, most of the studies did not find any significant changes in the cytokine profile [39]. The short half-life of cytokines, as well as their circadian rhythm, could influence these contrasting results. Furthermore, they are not reliable biomarkers, because they have auto and paracrine mechanisms of action. That is why they usually have a lower concentration in the peripheral blood than their local secretion sites. There is evidence that ME/CFS patients experience severe immunological changes during the first 3 years after the onset of symptoms [40]. Thus, the immune system of a patient that has experienced long-lasting ME/CFS could be vastly different when compared to a newly diagnosed one. Furthermore, it could be hypothesized that the excessive immune response in the first 3 years leads to the exhaustion of the cells in the later stages of the condition.

Reports about the state of B cells in ME/CFS patients are also enigmatic. While one study reported a higher proportion of naïve and transitional B cells and lower plasma blasts [33], another one demonstrated no significant difference in the plasma blasts but an elevation of memory B cells [34]. Two other studies found higher level of CD20+ CD5+ B cells [35,36]. This particular subset has innate-like functions and plays a key role in immune surveillance, homeostasis, clearance of self-antigens, and early defense against infections by producing natural antibodies (Nabs). Nabs have broad specificity and can bind a wide range of antigens with low affinity, including pathogen-associated molecular patterns (PAMPs) and self-antigens. They do not require prior exposure to specific antigens and are constantly secreted. By promoting the clearance of apoptotic cells and cellular debris, Nabs help maintain immune tolerance and prevent the accumulation of autoantigens [58]. Thus, disruptions in the balance of Nabs may contribute to the development of autoimmune diseases (Table 3) [15].

One double-blind, placebo-controlled clinical trial using Rituximab, which targets CD20+ B cells, achieved significant improvement in patients, with 67% reporting alleviated symptoms or complete clinical remission [59]. This could indicate that at least part of the ME/CFS patients have subtle alterations in their B-cell population associated with autoimmunity or the inability of the cells to exert their proper functions [2]. While Rituximab mostly targets and depletes B cells, it also reduces CD4+ effector cells and NK cells, TNF-α secretion, and promotes Treg function [59]. The beneficial action of Rituximab could be explained by the removal of autoreactive B cells, while allowing the regeneration of normal B-cell subpopulations. Furthermore, the depletion of B cells could potentially clear viruses like EBV, HHV-8, and CMV that use B cells as a reservoir [33]. Moreover, the induction of Treg anti-inflammatory and suppressive action by Rituximab could further explain the improvement in the patients. However, the six-year follow-up of the same patients in the Rituximab group showed no improvement in their condition [60]. Similarly, another randomized, placebo-controlled, double-blind trial does not associate Rituximab with clinical improvement in ME/CFS patients [61].

There are multiple studies reporting the existence of autoantibodies (AAbs) in ME/CFS patients targeting membrane and nuclear structures, as well as neurotransmitter receptors (Table 3) [2]. While some of these AAbs could be found in low concentrations in healthy individuals and their detection does not necessarily signify the patient has an autoimmune disease, their presence could reflect immune system overactivation or failure of immune tolerance mechanisms.

Data about the prevalence and type of autoantibodies in ME/CFS patients largely vary. According to five different studies, the presence of antinuclear antibodies (ANA) in ME/CFS patients varies between 13% and 68% [41,42,43,44,45]. Two studies reported that more than 90% of the patients had anti-cardiolipin Abs [46,47], whereas a third one found only 4% [48]. Abs against phospholipids, gangliosides, dsDNA, endothelial, and neuronal cells have also been reported [46,47,48,51]. Anti-phospholipid Abs could be associated with increased risk of clotting disorders and vascular abnormalities which could explain symptoms such as brain fog, headaches, and other neurological symptoms [62]. Additionally, Anti-endothelial cell antibodies (AECAs) are associated with an inability to regulate blood vessel dilation and constriction, possibly impairing the blood flow, oxygen delivery, and nutrient supply to tissues, possibly leading to the characteristic fatigue, cognitive dysfunction, and exercise intolerance seen in ME/CFS (Table 3) [50].

Muscle weakness, one of the most common symptoms in ME/CFS, was linked to the presence of anti- M1 muscarinic acetylcholine receptor (mAChR) Abs in patients [44]. Another study showed that these Abs functionally affect the central cholinergic system, and there is decreased binding of the mAChR in the brains of patients [49]. Abs against β2 adrenergic receptors (AdR) and M3/M4 mAChR were also reported [43]. Furthermore, higher levels of β2 AdR AAbs were linked to increased levels of anti-thyroid peroxidase Abs and ANA, as well as activation of HLA-DR+ T cells (Table 3) [2]. These receptors are involved in regulating autonomic functions, including cardiovascular responses, smooth muscle contraction, and metabolic regulation. Their disruption could potentially contribute to symptoms such as orthostatic intolerance, postural orthostatic tachycardia syndrome (POTS), and abnormal blood pressure regulation commonly observed in ME/CFS [2]. Oxidative and nitrosative stress are often described as crucial components of the possible pathogenic mechanisms causing ME/CFS. One study showed elevated levels of Abs against autoantigens that are released because of the damage caused by oxidative and nitrosative stress [63]. Interestingly, the severity of the symptoms was positively linked to higher levels of these AAbs [64].

However, several methodological and biological limitations currently constrain the interpretation of Aabs studies. Many studies rely on ELISA-based or cell-based assays that may detect low-affinity or low-avidity antibody binding, raising concerns about signal specificity. In some cases, measured signals may reflect background noise, cross-reactivity, or non-specific binding. Furthermore, the presence of an autoantibody does not necessarily imply pathogenic relevance. It is crucial for future studies to assess whether detected autoantibodies exert functional effects on receptor signaling, immune cell activity, or vascular responses. Without functional assays, it remains unclear whether reported autoantibodies are disease drivers, secondary epiphenomena, or incidental findings. AAbs detected in ME/CFS may reflect polyclonal immune activation rather than a stable autoimmune process. Such antibodies may be transient and fluctuate with immune activation, infection, or stress, limiting their utility as reliable biomarkers. Many reported autoantibody findings originate from single research groups and have not been consistently replicated by independent laboratories using standardized methods. Differences in antigen presentation, assay platforms, and analytical thresholds further complicate cross-study comparisons. Finally, positive findings are more likely to be published than null results. The under-reporting of negative studies may overestimate the prevalence and significance of autoantibodies in ME/CFS, complicating efforts toward biomarker validation.

Recently, a number of studies have linked certain genetic variants with a predisposition to autoimmunity and ME/CFS [2]. One study that performed high-resolution HLA genotyping found two independent alleles associated with ME/CFS [65]. The HLA class I allele C*07:04 could be associated with the dysregulation of CD8+ T cells and NK cells that is reported in some ME/CFS patients, whereas the HLA class II allele DQB1*03:03 could influence the interaction with CD4+ T cells. In certain autoimmune diseases, specific HLA class II alleles directly affect the acquired immune response via unique peptide-binding properties and HLA-TCR restriction [65]. It was found that those alleles are carried by approximately 10% of the patients and increase the risk of ME/CFS by 1.5–2-fold. According to the same study, ME/CFS patients carrying those specific risk alleles have a significantly higher autoimmune comorbidity. This further supports the theory about immune system engagement in the pathogenesis of ME/CFS [65].

While five Genome-Wide Association Studies (GWASs) of ME/CFS self-diagnosed patients included in the UK Biobank were performed, the different groups did not reach consensus, and their results are inconsistent [66]. In addition, two more groups undertook GWASs in different cohorts, and their results were once again conflicting. However, it is evident that ME/CFS is not a straightforward monogenic illness brought on by single nucleotide variations (SNVs), but it is most likely the result of complex interactions involving several genetic, epidemiological, and environmental components that are too intricate for GWAS-based methods to fully detect. More recently, one study found 199 single nucleotide polymorphisms (SNPs) that were highly related with 91% of ME/CFS patients, using a combinatorial analytics technique. These variations were linked to 14 genes that play key roles in stress, infection vulnerability, sleep disturbance, mitochondrial dysfunction, and autoimmune development [67]. The variations in those genes in ME/CFS patients could explain the complexity and heterogeneity of this condition.

Although the results about the state of the immune system are contradictory, there is a growing body of evidence that ME/CFS patients exhibit immunological, metabolic, and genetic changes that are in accordance with an autoimmune process.

## 5. Gastrointestinal Tract Dysbiosis

Many ME/CFS patients complain of gastrointestinal disturbances such as diarrhea, constipation, nausea, and vomiting [68,69], but the changes in the enteric microbiome have never been considered as an important criterion in the case definitions for ME/CFS diagnosis [70].

The intestinal tract hosts a large number of microorganisms that contribute to the regulation of local and systemic immunity, protection against pathogenic invaders, and even emotional well-being (through the “gut-brain axis”). Recent advancements in the field of sequencing technologies have allowed the examination of the exact composition of the gut microbiome in order to make associations between its changes and a variety of pathological conditions like obesity, inflammatory bowel diseases, and autoimmune and even neurologic disorders [71,72,73,74]. The most abundant microorganisms that comprise the gut microbiome are bacteria grouped within the following phyla: *Firmicutes*, *Bacteroidetes*, *Actinobacteria*, *Proteobacteria*, *Fusobacteria*, and *Verrucomicrobia*, among which *Firmicutes* and *Bacteroidetes* are the major types [75].

Any changes in the composition of the gut microbiome that are characterized by reduced diversity and imbalance between harmful and beneficial microorganisms are known as dysbiosis [76]. A growing number of studies show evidence of dysbiosis in ME/CFS patients, suggesting its role in disease pathogenesis, although a specific microbiome pattern has not been identified yet [20,74,77,78,79,80,81,82,83,84,85,86].

Inconsistencies in study design and differences in recruitment criteria and methods do not allow for statistically significant conclusions across the literature. For example, two studies reported a general decrease in bacterial abundance [82,86], whereas a third one reported an increase [23]. However, there is enough data proving that the intestinal microbiome is altered in ME/CFS patients. One of the most common findings between several studies is the decrease in anti-inflammatory species that belong to *Firmicutes* and a significant increase in the pro-inflammatory *Bacteriodetes* in stool samples of ME/CFS patients compared to healthy controls [74,78,83,84]. Another paper finds that there is an increase in potentially harmful bacteria that belong to *Enterobacteriaceae* [87]. Other researchers have found that there is a decrease in beneficial species such as *Faecalibacterium* [20,84,86] and *Bifidobacterium* [86]. *Faecalibacteruim prauznitzii*, for example, is known for its ability to produce butyrate, which is a shorth-chain fatty acid (SCFA) engaged in the maintenance of mucosal barriers and immunomodulation [88].

While the exact role of the gut microbiome in ME/CFS pathogenesis is still a matter of debate, and an exact bacterial taxonomic group cannot be directly used as a biomarker, there are several mechanisms related to the interplay between ME/CFS pathogenesis, symptomatology and the microbiome, among which intestinal barrier integrity and the “gut-brain axis” deserve particular attention [89,90].

It is generally thought that gut dysbiosis is one of the main causes of disturbed intestinal barrier integrity, which leads to increased gut permeability, also known as “leaky gut” [91]. In normal conditions, the intestinal barrier allows selective passage of substances and prevents translocation of gut microbes or their parts into the bloodstream, thus taking part in the processes of homeostasis [92]. It is hypothesized that SCFAs such as butyrate play a major function in maintaining healthy gut barrier, and the depletion of bacterial taxa with such properties (e.g., *Faecalibacteruim prauznitzii*, *Eubacterium rectale*) can be crucial for weakening that barrier [20]. Therefore, the loss of intestinal barrier integrity can be associated with bacterial translocation into the bloodstream followed by systemic inflammation generated via immune response towards bacterial toxins such as lipopolysaccharides (LPSs) derived from Gram-negative enterobacteria [93]. Evidence for such immune dysregulation can be supported by an altered cytokine profile in some ME/CFS patients, whose pro- as well as anti-inflammatory cytokine levels are reported to be elevated [91].

An intricate relationship between gut dysbiosis, intestinal permeability, chronic inflammation, and cognitive impairment in ME/CFS patients highlights the role of the so-called “gut-brain axis” in disease pathogenesis [92]. It is well established that the gastrointestinal system and the central nervous system have bidirectional communication executed through the following routes: the immune system pathway by cytokines; the hormonal route by the following neurotransmitters: Gamma-aminobutyric acid (GABA), serotonin or dopamine; neural routes including the vagus nerve and spinal pathway; and the metabolic pathway including SCFAs [16]. The disrupted gut–brain communication in ME/CFS involves altered neurochemical signaling, immune responses, and neuronal health [94]. Key pathomechanisms focus on abnormal tryptophan metabolism, reduced SCFA levels, D-lactic acidosis, and modified kynurenine pathway activity [16]. The bidirectional nature of this axis means gut dysfunction can influence central nervous system inflammation and cognitive symptoms [95,96].

Although specific microbial taxa cannot yet be considered diagnostic biomarkers, convergent evidence supports gut dysbiosis as a contributor to systemic immune activation. Effect sizes for reduced butyrate-producing taxa and increased pro-inflammatory species are modest but consistent across independent cohorts, supporting biological relevance despite inter-study variability.

## 6. Neuroendocrine Interactions

The bidirectional communication between the immune and endocrine systems is thought to be central to the complex pathophysiology of ME/CFS [97]. Cytokine imbalance can disrupt the hypothalamic–pituitary–adrenal (HPA) axis, a vital neuroendocrine system that controls the body’s stress response. This disruption can lead to changes in hormone production, which exacerbates immune dysregulation and contributes to the debilitating symptoms seen in ME/CFS patients. In addition, the chronic inflammation associated with ME/CFS can affect the endocrine system by disrupting the normal function of hormone-producing glands such as the thyroid and gonads. This can manifest itself in a wide range of symptoms, including fatigue, cognitive impairment, sleep disturbances, and hormonal imbalances [98,99].

Thyroid hormones, triiodothyronine (T3) and thyroxine (T4), are essential for maintaining a healthy and balanced metabolism. They play a key role in regulating various bodily functions such as energy production, temperature regulation, and cognitive function [91]. The thyroid function in some cases of ME/CFS is impaired, including subclinical hypothyroidism and altered thyroid hormone conversion. Subclinical hypothyroidism can lead to a decrease in overall metabolic activity, resulting in symptoms such as fatigue, weight gain, cold intolerance, and cognitive impairment. On the other hand, altered thyroid hormone conversion can result in a relative T3 deficiency even when T4 levels appear normal, contributing to the complex array of symptoms experienced by people with ME/CFS [100,101].

ME/CFS has multiorgan repercussions but tends to have pathology framed around neurological, immune and endocrine complications and is often referred to as a neuroimmune-endocrine dysfunction, with an exclusively clinical diagnosis [102].

Detailed observations of clinical conditions with low levels of circulating cortisol, distinguished by devitalizing fatigue, lead to increased interest in the role of the HPA axis in ME/CFS [97]. Therefore, along with additional symptoms including arthralgia, myalgia, sleep disturbance, and mood instability that are often present in ME/CFS, Addison’s disease, glucocorticoid withdrawal, and bilateral adrenalectomy are also linked to fatigue [103,104]. These findings led to the theory that low cortisol levels in the bloodstream are a contributing factor to the weariness seen in ME/CFS patients [97].

Investigation of HPA axis dysfunction in patients with ME/CFS has shown conflicting results in the literature. The most consistent finding, however, is a low salivary cortisol-awakening response in adult ME/CFS patients [105,106]. Further evidence, including aberrant diurnal hormone variation, low baseline levels of HPA axis hormones, enhanced sensitivity to glucocorticoids, and reduced HPA axis response to physical and psychological stressors are presented as symptoms in people who suffer from ME/CFS [107]. Nevertheless, there is not any solid proof that any disruption in the HPA axis is unique to ME/CFS or that it causes the illness itself instead of just being one of its many side effects or comorbidities [97].

One explanation of the low cortisol levels observed in ME/CFS patients is that they could be related to dysregulation of the stress response [108]. This hypothesis posits that HPA axis hypofunction is the result of a “stressed crash” or “exhaustion” phenomenon, in which a prolonged period of stress induces HPA axis hyperfunction, which would then transition into HPA axis hypofunction. Persistent stressors can exhaust the body’s response, leading to HPA axis loss of stress-coping ability beyond decreased cortisol output [97,109]. One such example is hypothesized in one study where salivary melatonin levels were measured, and contrary to the expectations (linked to sleep disturbances, observed in ME/CFS), those levels were elevated in patients with ME/CFS. This might represent a marker of increased susceptibility to stress-induced hypothalamic disruption [110]. In individuals with ME/CFS, there is a linear relationship between these two hormones because adrenocorticotrophic hormone primarily regulates plasma cortisol [111,112]. Recently, families with a high frequency of ME/CFS have been found to have mutations in the cortisol-binding globulin (CBG) gene. Despite the normal 24 h urine-free cortisol excretion, the substantially decreased cortisol-binding ability in these individuals probably leads to insufficient transport of cortisol to the target organs, resulting in a condition of functional hypocortisolism. It is important to conduct more research to find out how CBG anomalies relate to ME/CFS [113]. In addition, in one study the antidiuretic hormone (ADH) is downregulated, which further supports the concept of central neuroendocrine involvement in ME/CFS and complements existing evidence of HPA axis dysfunction [114].

Multiple reports exist that depict correlations between hormones of the HPA axis, the thyroid system, and the sympathetic/adrenal medulla system in ME/CFS patients [112,115]. Furthermore, it is well known that HPA axis hormones and glucocorticoids also affect the immune system [116]. On one hand, the HPA axis hormones can tilt the immune response toward Th2, and on the other the Th1 response stimulates HPA axis hormone production. Certain studies that correlate immune system cell activity with thyroid, sex hormone, and HPA axis state indicate the potential loss of thyroid function in ME/CFS, which is worsened by HPA axis inactivity, and suggest the cause of persistent inflammation [117].

There are several reasons to investigate the growth hormone (GH) axis in ME/CFS. Firstly, there are congruent findings in the literature on fibromyalgia, wherein sleep difficulties and muscular pain have been associated with reduced GH function [104]. Two studies delved into the growth hormone-insulin-like growth factor (GH-IGF) system in ME/CFS patients. The first study found no significant GH function differences [118], whereas the second study identified reduced nocturnal GH release and diminished GH response to the IST (insulin stress test) in ME/CFS [119]. However, IGF (insulin-like growth factor) levels remained unchanged, and responses to other challenges were unaffected.

Even with extensive scientific attention, the origin of the illness remains unidentified, with most studies indicating a multifaceted cause. The prevailing belief is that the emergence and persistence of ME/CFS results from a complex interaction involving neuroendocrine, humoral, immunological, and autonomic nervous system disorders, along with a psychological predisposition.

## 7. Metabolic Impairments

The presence of symptoms like post-exertional malaise (PEM) and persistent fatigue that is not significantly relieved by rest in ME/CFS has led researchers to explore mitochondrial function and cellular energy metabolism, as impairments in these areas may play a role in the pathology of the syndrome [120]. A growing body of research has revealed a connection between mitochondrial dysfunction (oxidative stress, redox imbalance, disrupted calcium regulation, reduced adenosine triphosphate (ATP) production, and changes in mitochondrial membrane potential and permeability) and persistent low-grade systemic inflammation in individuals with ME/CFS [21]. Deficient ATP production was demonstrated in multiple studies, which could explain the wide variety of multisystemic symptoms (PEM, fatigue, headaches, myalgia, arthralgia, and sleep and cognitive dysfunction) [21,22]. Moreover, one study showed insufficient ATP synthesis specifically by mitochondrial complex V, which is the final step of oxidative phosphorylation, alongside elevated function of complexes I-IV and activity of TORC1 (Target of Rapamycin Complex I). TORC1 plays an important role in energy homeostasis and mitochondrial function by sensing changes in environmental factors [121]. It was suggested that the deficiency of ATP could be compensated by the elevation of the other two. However, the authors hypothesize that this chronic upregulated state of complexes I-IV and TORC1 is only sufficient to meet the basic needs of the cells and not enough to provide for a further increase in ATP demand, for example, during and post-exercise [121]. Findings from another study further indicate that there is a compensatory shift from conventional glycolysis required for mitochondrial respiration to alternative pathways like fatty acid β-oxidation. However, it is suggested that this change is not mediated by a decrease in glycolysis but by an increase in alternative catabolic pathways [122].

One circulatory metabolomics study found that patients with ME/CFS compared to HCs exhibit lower levels of acylcholines, dipeptides, and three classes of steroids, alongside elevated levels of sphingolipids [123]. Long-chained acylcholines are involved in blood pressure regulation and hydrochloric acid secretions in the gut; thus, a decrease in those compounds could potentially explain the orthostatic intolerance and leaky gut symptoms experienced by some patients [123]. In addition, dipeptides, once thought to be merely byproducts of protein metabolism, are now recognized for their roles in digestion, energy production, cell signaling, tissue repair, and hormone regulation [124]. Therefore, their diminished presence in ME/CFS patients might contribute to disruptions across these biological functions. However, dipeptides remain relatively understudied, and their correlation to ME/CFS should be further evaluated [123]. Moreover, the reduced levels of certain steroid classes observed in patients may be indicative of HPA axis dysfunction, as previously discussed [123]. On the other hand, bioactive sphingolipids form extremely complex networks that are associated with almost all cellular processes including cell growth, cell cycle, apoptosis, inflammation, stress response, and cell adhesion and migration [125]. While Germain et al. reported that ME/CFS patients had an increase in those compounds, Naviaux and colleagues observed the contrary [123,126]. Although the functions of sphingolipids in pathological contexts are still severely misunderstood and the research findings are inconsistent, it is safe to assume that their dysregulation could be involved in the disruption of some of the above-mentioned cellular processes [125].

## 8. Endothelial Dysfunction

Oxidative stress and nitrosative stress are integral to the pathophysiology of endothelial dysfunction in ME/CFS. Oxidative stress can be defined as an imbalance between the production of ROS (reactive oxygen species) and the body’s antioxidant defense mechanisms. In ME/CFS, this imbalance can be further exacerbated by several factors, including mitochondrial dysfunction, immune system dysregulation, and chronic inflammation [127,128]. ROS can directly damage endothelial cells by oxidizing lipids, proteins, and DNA, which in turn leads to endothelial dysfunction [129,130].

Similarly, nitrosative stress is characterized by an excess of reactive nitrogen species (RNS), including nitric oxide (NO), peroxynitrite (ONOO-), and nitrogen dioxide (NO_2_). These molecules can react with cellular components, including proteins and lipids, causing nitrosylation and nitration, which disrupt endothelial cell function. In ME/CFS, elevated levels of nitric oxide synthase (NOS) and inducible nitric oxide synthase (iNOS) may contribute to increased production of nitric oxide and peroxynitrite, further promoting nitrosative stress and endothelial dysfunction [129,131,132].

Both oxidative and nitrosative stress can induce inflammatory responses within the walls of blood vessels, characterized by the upregulation of endothelial cell adhesion molecules (CAMs), including intercellular adhesion molecule-1 (ICAM-1) and vascular cell adhesion molecule-1 (VCAM-1) [132]. The CAMs facilitate the adhesion and transmigration of immune cells, including monocytes and lymphocytes, into the vessel wall, perpetuating vascular inflammation and dysfunction [133,134]. Oxidative stress can deplete nitric oxide bioavailability by reacting with NO, forming peroxynitrite. This not only impairs endothelial-dependent vasodilation but also exacerbates oxidative damage to endothelial cells. The depletion of nitric oxide contributes to vasoconstriction, platelet aggregation, and leukocyte adhesion, all of which contribute to endothelial dysfunction [130].

As mentioned above, autoantibodies against β2-adrenergic receptors and M3 acetylcholine receptors have been identified in some individuals with ME/CFS. These autoantibodies have the potential to disrupt the normal functions of the nervous system. Both ß2AdR and M3 acetylcholine receptors are important vasodilators. Consequently, it can be anticipated that their functional disturbance will result in vasoconstriction and hypoxemia [135,136,137].

The clinical manifestations of endothelial dysfunction ME/CFS may vary but often include symptoms related to impaired blood flow regulation and vascular tone. Patients may present with persistent fatigue, cognitive impairment, and orthostatic intolerance, which is characterized by symptoms such as dizziness, lightheadedness, and palpitations upon standing. Furthermore, endothelial dysfunction may also contribute to the development of exercise intolerance, muscle pain, and headaches in individuals with ME/CFS. Endothelial dysfunction may increase the risk of developing cardiovascular complications, including hypertension, coronary artery disease, and microvascular dysfunction [135,136,137,138,139]. It is of utmost importance to gain an understanding of the underlying endothelial dysfunction in ME/CFS to effectively manage symptoms and reduce the risk of associated cardiovascular complications.

## 9. Conclusions

Myalgic encephalomyelitis/chronic fatigue syndrome is a multifactorial condition with complex pathophysiology involving dysfunction across several key biological systems. Increasing evidence points to significant disturbances in immune function, with abnormalities in the function and phenotype of NK cells, T cells, and B-cell populations, often accompanied by autoantibody production. Furthermore, specific HLA variants and the SNPs found from GWAS studies could be associated with a higher risk of developing ME/CFS.

The gastrointestinal tract also plays a critical role, with studies showing that gut dysbiosis, increased intestinal permeability, and reduced levels of short-chain fatty acids contribute to systemic inflammation and immune activation. The gut–brain axis emerges as a crucial mediator, linking microbiome imbalances to neurocognitive and fatigue symptoms through immune, neural, and metabolic pathways. However, alterations of the gut microbiome reported in ME/CFS are not disease-specific, as similar patterns have been observed in a variety of chronic inflammatory, metabolic, and neuroimmune disorders. Consequently, gut dysbiosis should not be interpreted as a diagnostic biomarker for ME/CFS but rather as a context-dependent modifier of immune and metabolic function. Importantly, comparable microbial changes may give rise to distinct disease phenotypes in different individuals, depending on host-specific factors such as genetic background, immune tone, epithelial barrier integrity, neuroendocrine regulation, and prior environmental exposures. The immune system plays a central role in shaping host–microbe interactions through recognition of microbial-associated molecular patterns, regulation of mucosal barrier function, and responses to microbial metabolites such as SCFAs, which influence T-cell differentiation and regulatory T-cell function. In ME/CFS, immune dysregulation may both drive and result from microbiome alterations, suggesting a bidirectional relationship that contributes to disease heterogeneity and chronic immune activation rather than a unidirectional causal mechanism.

Neuroendocrine abnormalities, particularly dysfunction of the HPA axis, further compound the clinical picture. Hypocortisolism, altered thyroid hormone metabolism, and possible disruptions in the growth hormone axis have all been reported, suggesting that hormonal imbalances perpetuate both immune dysfunction and metabolic disturbances.

Metabolic impairments, most notably mitochondrial dysfunction and inefficient ATP production, likely underpin the hallmark symptom of post-exertional malaise. Compensatory shifts in energy metabolism and alterations in lipid and steroid profiles suggest systemic bioenergetic failure, which correlates with the severity and persistence of fatigue and cognitive dysfunction.

Finally, endothelial dysfunction, driven by oxidative and nitrosative stress, contributes to impaired vascular regulation. This may explain the frequent findings of orthostatic intolerance, reduced tissue perfusion, and increased cardiovascular risk in ME/CFS patients. The presence of autoantibodies targeting vasoregulatory receptors reinforces the link between immune dysregulation and vascular pathology.

**Figure 2:**
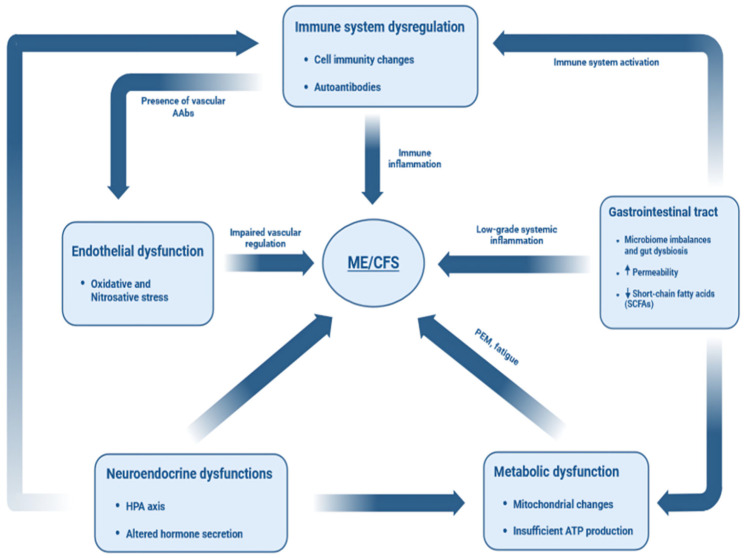
Summary of conceptual integrative framework of the intersecting systemic alterations leading to the symptoms observed in ME/CFS patients.

Collectively, these dysfunctions appear to arise from a common underlying process, which is chronic inflammation driven by immune dysregulation and disturbance of the neuroendocrine–immune axis. This state may be triggered by infectious agents or prolonged physiological stress, further influenced by genetic predisposition and gut microbiome imbalance. The consequent oxidative and nitrosative stress contributes to widespread multisystem impairment, which in turn exacerbates immune dysfunction, thereby establishing a self-sustaining pathological cycle (Figure 2).

In summary, ME/CFS represents a systemic disorder arising from the intersection of immune, gastrointestinal, neuroendocrine, metabolic, and vascular dysfunction. The proposed cycle represents a conceptual integrative framework, grounded in convergent evidence from multiple biological domains, rather than a quantitatively validated causal model. The inconsistent results across studies are probably caused by differences in diagnostic criteria, cohort size, disease duration, analytical techniques, and biological sample types. Other factors that might influence the heterogeneity of the findings are the temporal evolution of ME/CFS, particularly differences between early and long-standing disease, and severity-based subgroups. Thus, this review highlights the need for more precise patient stratification and unified diagnostic criteria to improve cohort comparability and unravel the complex mechanisms underpinning this debilitating condition, as well as longitudinal studies combining multi-omics, clinical phenotyping, and functional assays to provide quantitative validation of these interactions.

## Figures and Tables

**Table 1:** Chronological order of diagnostic criteria used for ME/CFS.

Year/Name of Criteria	Core Requirements	Duration of Symptoms	Additional Notes	References
1988—CDC (Holmes)	Debilitating fatigue + exclusion of other causes	≥6 months	Diagnosis requires ≥ 8 of 11 symptoms (fever, sore throat, lymphadenopathy, myalgia, insomnia, etc.) or 6 symptoms + ≥2 physical findings (fever, pharyngitis, tender nodes)	[4]
1991—Oxford	Fatigue as main symptom, definite onset, debilitating, present ≥ 50% of time	≥6 months	Includes psychiatric conditions; recognizes post-infectious subtype	[5]
1994—CDC (Fukuda)	Chronic fatigue (unexplained, disabling, not lifelong, not relieved by rest) + ≥4 of memory/concentration problems, sore throat, lymphadenopathy, myalgia, arthralgia, new headaches, unrefreshing sleep, PEM > 24 h	≥6 months	Widely used in research; requires exclusion of psychiatric and medical causes	[6]
1994—London Criteria	Fatigue triggered by exertion, impaired short-term memory/concentration, fluctuating symptoms	≥6 months	Autonomic/immune symptoms common; initially research-oriented	[7]
2003—Canadian Consensus	Severe fatigue, PEM/fatigue, sleep dysfunction, myalgia, ≥2 neuro/cognitive symptoms, + ≥1 symptom from ≥2 of autonomic, neuroendocrine, immune	≥6 months	Excludes primary psychiatric illness; 2010 revision added functional impairment thresholds	[8]
2011—International Consensus (ICC)	Post-exertional neuroimmune exhaustion (PENE) + ≥1 neurological, ≥3 immune/GI/GU, ≥1 energy production symptom	No duration requirement	Focused on biological abnormalities; defines severity levels (mild → very severe)	[9]
2015—Institute of Medicine (IOM)	Fatigue, PEM, unrefreshing sleep + (cognitive impairment or orthostatic intolerance)	≥6 months; symptoms ≥ 50% of time with moderate severity	Simplified for clinical use; CDC currently adopts this	[10]
2021—UK NICE Guideline	Fatigue, PEM, unrefreshing/disturbed sleep, cognitive difficulties	≥6 weeks (adults), ≥4 weeks (children)	Practical clinical guideline; emphasizes early recognition	[11]

**Table 2:** Viral agents associated with ME/CFS etiology.

Virus	Evidence/Association	Proposed Mechanism	References
Epstein–Barr virus (EBV)	Frequently reported in onset cases; serological evidence of reactivation	Latent infection in B cells, molecular mimicry, autoimmunity trigger	[12,13,14]
Human Herpes viruses (HHV-6A, HHV-6B, HHV-7, HHV-8)	Detected in tissues/sera of ME/CFS patients	Latency/reactivation, immune dysregulation	[12,13]
Cytomegalovirus (CMV)	Reported in subsets of patients	Chronic infection, immune exhaustion	[12,13]
Human Parvovirus B19	Linked to ME/CFS onset in case studies	Autoimmunity trigger via tissue damage and antigen release	[12,13]
Enteroviruses	Historical outbreaks associated with CFS-like illness	Persistent infection, chronic inflammation	[12]
Retroviruses (XMRV, others)	Early studies suggested association, later disputed	Chronic immune activation	[12]
Ross River virus	Post-viral fatigue syndrome documented after outbreaks	Post-viral immune dysregulation	[12]
Coronaviruses (incl. SARS-CoV-2)	Post-COVID-19 syndrome overlaps with ME/CFS phenotype	Persistent immune activation, autoimmunity	[12,13]

**Table 3:** Alterations of the immune system in ME/CFS patients.

Immune Component	Reported Alteration	Clinical/Functional Impact	References
NK cells	↓ Cytotoxic activity; altered phenotype (↓ perforin, granzyme; impaired MAPK and Ca^2+^ signaling)	Reduced viral clearance, chronic infection risk	[23,24,25]
CD8+ T cells	Exhaustion phenotype (↑ PD-1, CTLA-4, TIGIT; ↓ proliferation, ↓ mitochondrial potential)	Impaired viral control, chronic inflammation	[26,27,28]
CD4+ T cells	↓ Glycolysis, ↑ fatty acid oxidation	Metabolic dysfunction, immune exhaustion	[27,28]
Regulatory T cells (Tregs)	Conflicting: ↑ count in some studies, ↓ in others	Dysregulated tolerance, possible autoimmunity	[29,30,31,32]
B cells	↑ Naïve and transitional B cells; ↑ CD20^+^CD5^+^ subset	Impaired antibody regulation, autoantibody production	[33,34,35,36]
Cytokines	Contradictory reports: Th2 skewing in some studies, mixed Th1/Th2 in others	Heterogeneous immune activation states	[37,38,39,40]
Autoantibodies–ANA	Varying prevalence in different studies (13–68%)	Non-specific marker of autoimmunity	[41,42,43,44,45]
Anti-cardiolipin antibodies	Varying prevalence in different studies (4–90%)	Thrombosis, vascular abnormalities	[46,47,48]
Anti-β2 adrenergic receptor (AdR) Abs	Detected in patients	Orthostatic intolerance, POTS, BP dysregulation	[43]
Anti-muscarinic AChR (M1, M3, M4) Abs	Detected in multiple cohorts	Muscle weakness, cholinergic dysfunction	[44,49]
Anti-endothelial cell Abs (AECA)	Detected in patients	Vascular dysregulation, hypoxia, cognitive dysfunction	[50]
Other autoantibodies (dsDNA, gangliosides, phospholipids)	Varying prevalence in different studies	Possible neurological and vascular symptoms	[46,47,48,51]

Note: ↓–decreased; ↑–increased.

## Data Availability

No new data were created or analyzed in this study. Data sharing is not applicable to this article.

## Abbreviations

The following abbreviations are used in this manuscript:

AAbs	Autoantibodies
Abs	Antibodies
AdR	Adrenergic Receptor
AECAs	Anti-Endothelial Cell Antibodies
ANA	Antinuclear Antibodies
APC	Antigen-Presenting Cell
Ca^2+^	Calcium Ion
CAMs	Cell Adhesion Molecules
CBG	Cortisol-Binding Globulin
CDC	Centers for Disease Control and Prevention
CD	Cluster of Differentiation
CMV	Cytomegalovirus
dsDNA	Double-Stranded DNA
EBV	Epstein–Barr Virus
FAO	Fatty Acid Oxidation
GABA	Gamma-Aminobutyric Acid
GH	Growth Hormone
GWAS	Genome-Wide Association Study
HCs	Healthy Controls
HHV	Human Herpes Virus
HLA	Human Leukocyte Antigen
HPA	Hypothalamic–Pituitary–Adrenal Axis
ICC	International Consensus Criteria
ICAM-1	Intercellular Adhesion Molecule 1
IGF	Insulin-Like Growth Factor
IL	Interleukin
iNOS	Inducible Nitric Oxide Synthase
IOM	Institute of Medicine
IST	Insulin Stress Test
LPS	Lipopolysaccharide
mAChR	Muscarinic Acetylcholine Receptor
MAPK	Mitogen-Activated Protein Kinase
ME/CFS	Myalgic Encephalomyelitis/Chronic Fatigue Syndrome
Nabs	Natural Antibodies
NK	Natural Killer (Cells)
NO	Nitric Oxide
NO_2_	Nitrogen Dioxide
ONOO^−^	Peroxynitrite
PAMPs	Pathogen-Associated Molecular Patterns
PEM	Post-Exertional Malaise
POTS	Postural Orthostatic Tachycardia Syndrome
RNS	Reactive Nitrogen Species
ROS	Reactive Oxygen Species
SCFAs	Short-Chain Fatty Acids
SNPs	Single-Nucleotide Polymorphisms
SNVs	Single-Nucleotide Variants
T3	Triiodothyronine
T4	Thyroxine
TCR	T-Cell Receptor
TORC1	Target of Rapamycin Complex 1
Treg	Regulatory T Cell
VCAM-1	Vascular Cell Adhesion Molecule 1
